# FRET-Based Ca^2+^ Biosensor Single Cell Imaging Interrogated by High-Frequency Ultrasound

**DOI:** 10.3390/s20174998

**Published:** 2020-09-03

**Authors:** Sangpil Yoon, Yijia Pan, Kirk Shung, Yingxiao Wang

**Affiliations:** 1 Department of Aerospace and Mechanical Engineering, University of Notre Dame, Notre Dame, IN 46556, USA; 2 Department of Bioengineering, University of California, San Diego, CA 92092, USA; panyijia07@gmail.com (Y.P.); yiw015@eng.ucsd.edu (Y.W.); 3 Department of Biomedical Engineering, University of Southern California, Los Angeles, CA 90089, USA; kkshung@usc.edu

**Keywords:** fluorescence resonance energy transfer (FRET), EGFP and FusionRed FRET pair, dual FRET imaging, high frequency ultrasound, single cell stimulation/intracellular delivery

## Abstract

Fluorescence resonance energy transfer (FRET)-based biosensors have advanced live cell imaging by dynamically visualizing molecular events with high temporal resolution. FRET-based biosensors with spectrally distinct fluorophore pairs provide clear contrast between cells during dual FRET live cell imaging. Here, we have developed a new FRET-based Ca^2+^ biosensor using EGFP and FusionRed fluorophores (FRET-GFPRed). Using different filter settings, the developed biosensor can be differentiated from a typical FRET-based Ca^2+^ biosensor with ECFP and YPet (YC3.6 FRET Ca^2+^ biosensor, FRET-CFPYPet). A high-frequency ultrasound (HFU) with a carrier frequency of 150 MHz can target a subcellular region due to its tight focus smaller than 10 µm. Therefore, HFU offers a new single cell stimulations approach for FRET live cell imaging with precise spatial resolution and repeated stimulation for longitudinal studies. Furthermore, the single cell level intracellular delivery of a desired FRET-based biosensor into target cells using HFU enables us to perform dual FRET imaging of a cell pair. We show that a cell pair is defined by sequential intracellular delivery of the developed FRET-GFPRed and FRET-CFPYPet into two target cells using HFU. We demonstrate that a FRET-GFPRed exhibits consistent 10–15% FRET response under typical ionomycin stimulation as well as under a new stimulation strategy with HFU.

## 1. Introduction

**Figure 1:**
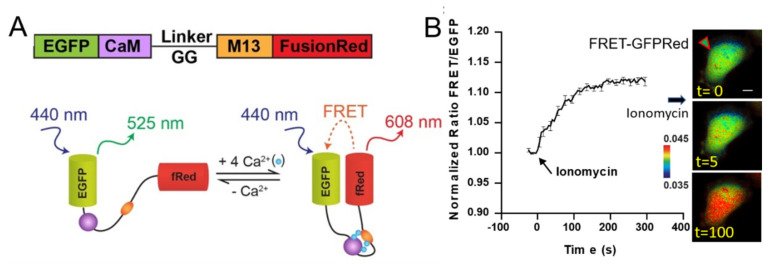
(**A**) A schematic diagram of a new fluorescence resonance energy transfer (FRET) biosensor (FRET-GFPRed). A new FRET-based Ca^2+^ biosensor with EGFP and FusionRed fluorophores (FRET-GFPRed) was designed to show FRET between EGFP and FusionRed. FRET increases when calcium ions bind to Calmodulin (CaM) and M13, which provides dynamic readouts of intracellular calcium concentration. (**B**) Time course of the FRET/EGFP signal ratio. FRET-GFPRed expressing HeLa cells were stimulated with ionomycin. Images on the left represents before (t = 0 s), 5 s, and 100 s after ionomycin stimulation (1 μM) in HeLa cells at t = 0 s. A green arrowhead surrounded by red lines indicates HeLa cells transfected with FRET-GFPRed. A scale bar represents 10 μm. Error bars indicate ± one SEM.

Calcium is an important signaling molecule, playing a significant role in neuronal function [1,2], determining cell development [3,4,5], controlling synaptic plasticity [6], and regulating tumor progression [7]. Therefore, the intracellular concentration of Ca^2+^ is tightly regulated and the precise measurements of spatiotemporal dynamics of Ca^2+^ may unveil crucial biochemical activities. Numerous Ca^2+^ indicators have been developed by many research groups. Synthetic fluorescent chelators were used to measure intracellular Ca^2+^ concentration [8,9,10]. Chelators are easy to image but they are not targetable to specific organelles and less bright. To address this, a genetically encoded fluorescence resonance energy transfer (FRET)-based Ca^2+^ biosensor (BS) was developed using donor and acceptor fluorescent proteins, Calmodulin (CaM), and CaM-binding peptide M13 [11]. As Figure 1A describes, four Ca^2+^ bind to CaM, which induces conformational changes to a compact form. The relative distance and orientation of two fluorophores decrease, resulting in FRET increase. FRET BS has been revolutionized many fields including Ca^2+^ signaling, caspase activation, and in vivo optogenetics [12,13]. Since then, FRET Ca^2+^ live cell imaging has advanced [14]. Single fluorescent protein (FP)-based genetically encoded intracellular Ca^2+^ indicators have also been widely used such as GCaMP [15]. Further advances on the development of single fluorescent protein-based indicators have been achieved. For example, GCaMP6 has increased sensitivity and fast kinetics [16], and RCaMP [17] and R-GECO1 [18] are compatible with other reporter constructs with GFP signal.

FRET-based biosensors have revolutionized the field of live cell imaging. As a noninvasive spectroscopic technique, FRET-based BS can dynamically monitor conformational changes between donor and acceptor fluorophores, which enables the visualization of signaling events in live cells with high spatiotemporal resolution. FRET-based live cell imaging has advantages over single fluorescent protein-based indicators because the FRET changes are the ratio of FRET (acceptor) intensity to the donor intensity. Cells with FRET-based BS express strong fluorescence signal at donor fluorophore’s wavelength. FRET-based BS has a high signal-to-noise ratio and is suitable for long-term imaging sessions experiments, because the ratiometric nature of FRET-based live cell imaging decreases the influence of changes of cell shape and excitation light fluctuations [14,19]. Particularly, the development of FRET-based Ca^2+^ BS has been actively pursued because of the importance of Ca^2+^ as already described. Yellow cameleon version 3.60 (YC3.60) outperforms other versions of FRET-based Ca^2+^ BS [20]. Including YC3.60, enhanced cyan and yellow fluorescent proteins (ECFP/YFP) has been the most common and sensitive donor and acceptor fluorophore pairs for many FRET-based BS [11,19,21,22]. Due to this reason, there haves been difficulties in monitoring the intracellular concentration of Ca^2+^ while visualizing other molecular events such as Src and Fyn using dual FRET live cell imaging. Here, to address this, we have developed a new FRET-based Ca^2+^ BS with an EGFP and FusionRed [23] pair (FRET-GFPRed) to realize dual FRET live cell imaging for simultaneous monitoring of Ca^2+^ and other molecular events such as Src with high temporal resolution.

Currently, chemical stimulation has been widely used to induce molecular events that can be detected by FRET BS. However, chemical stimulation affects whole populations of cells in a dish, and is very difficult to induce subcellular stimulation within cells and trigger repeated stimulations to the same cell. Previously, to apply mechanical force, optical tweezers were used to transmit trapping force into a cell or subcellular locations by coupling a bead and a cell surface [19,24,25]. However, the apparatus for realizing optical tweezers is very bulky and expensive. Beads provide another complication to this method. To address these difficulties, we have developed a 150 MHz high-frequency ultrasound (HFU) to directly manipulate target single cells without beads or microbubbles. Electronics to control HFU are much cheaper than components of laser-optics. Single cell targeting and manipulation can be realized by HFU due to its focusing ability. When the frequency of HFU exceeds 150 MHz, the size of the focal area approaches that of a subcellular region smaller than 10 μm [26,27]. HFU can be used to mimic the physiological cues that trigger changes in gene expression and cell behavior. For example, HFU can serve to generate microstreaming around cells, to stretch a cell’s lipid bilayer, and to push cell membranes directly for an extended time without coming into contact with the cells. Different types of cells experience different mechanical stimuli. Red blood cells, endothelial cells, and somatosensory neurons experience various types of forces during circulation [28], blood flow-induced shear stress [29], and tactile stimuli [30], respectively. Therefore, HFU provides mechanical stimuli to activate the release of Ca^2+^ from endoplasmic reticulum within the cell or introduce Ca^2+^ via mechanotransduction ion channels. Direct disruption of cell plasma membrane by HFU can introduce Ca^2+^ and DNA or protein for the intracellular delivery [26,27]. Because disrupting cell plasma membrane does not occur in real situation, we will further improve our technique to directly stimulate organelles and induce channel opening via HFU without disruption in the future. 

In this study, we have combined dual FRET-based live cell imaging, single cell level stimulation and the intracellular delivery of FRET BS using HFU. We demonstrated that the new FRET-based Ca^2+^ BS, which was developed by combining EGFP and FusionRed (FRET-GFPRed), can offer distinct and consistent FRET response compared to a well-known FRET-based Ca^2+^ BS with ECFP/YPet (YC3.6 FRET Ca^2+^ biosensor [14], FRET-CFPYPet) under various conditions. We also tested the precise spatial control of stimulating single cells and the delivery of the developed FRET BS using HFU.

## 2. Materials and Methods

### 2.1. Plasmids and Constructs

The calcium biosensor YC3.60 with ECFP and YPet was developed previously [20]. Underlined sequences indicate BsaI restriction sites. Calmodulin (CaM) and M13 were amplified by PCR using sense 5′-ATAGGTCTCACAAGAGCGGTATGCATGACCAACTGACAGAAG-3′ and antisense primers 5′-AAATGGTCTCAGCATGCCAGTGCCCCGGAGCTGGAGATCT-3′. The EGFP fragment was amplified by PCR using primer pair forward 5′-AAATGGTCTCA***ATG***CATGGTGAGCAAGGGCGAGGAGCT-3′ (start codon bold/italic) and reverse 5′-AAATGGTCTCACACTTCACTTGTACAGCTCGTCCATGCCG-3′. The FusionRed fragment was also amplified by PCR using primer pair forward 5′-AAATGGTCTCAATGCATGGTGAGCGAGCTGATTAAGG-3′ and reverse 5′-AAATGGTCTCACACT***TCA***TTTACCTCCATCACCAGCCCC-3′ (stop codon bold/italic). The PCR products were cloned into pCDNA3.1 (Thermo Fisher Scientific, Waltham, UK) between two BsaI restriction sites to generate the new FRET-GFPRed calcium biosensor as shown in Figure 1A.

### 2.2. Ultrasound Stimulation and Intracellular Delivery of FRET Biosensors Using High Frequency Ultrasound

**Figure 2:**
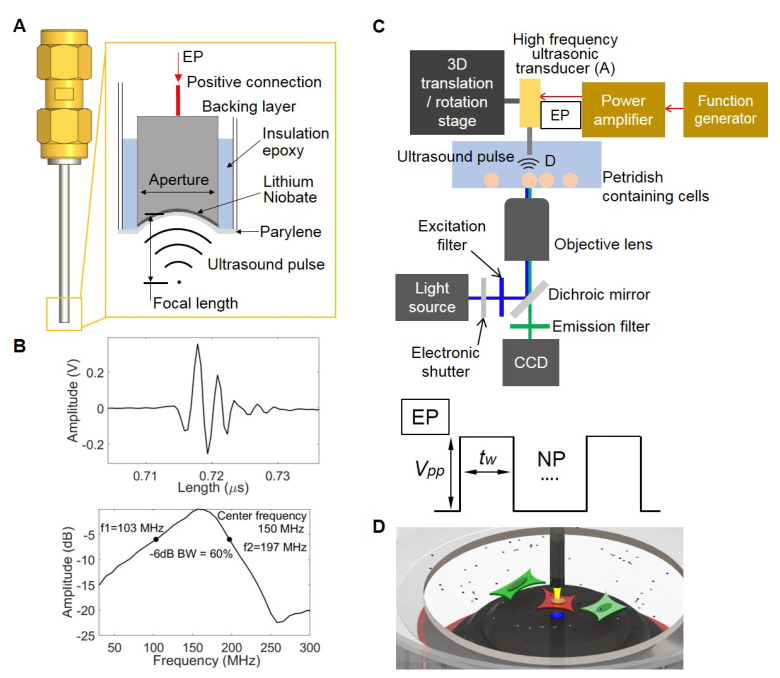
High-frequency ultrasonic transducer and integrated system for the intracellular delivery and stimulation. (**A**) Schematic diagram of 150 MHz ultrasonic transducer shows SMA connector (yellow part) and needle type transducer (grey part) for cell manipulation. Lithium niobate (LibNO_3_) was used to generate 150 MHz ultrasound pulse for the intracellular delivery and stimulation. Aperture (1 mm) was focused to have 1 mm focal depth with fnumber of 1. Electrical pulse (EP) was applied through positive connection to trigger LibNO_3_. (**B**) Experimentally measured echo and its spectrum of the developed transducer shows that actual center frequency was 150 MHz. (**C**) An integrated system for the intracellular delivery and stimulation of target cells was comprised of a fluorescence microscope and a high frequency ultrasound transducer shown in A. A function generator and a power amplifier generated EP to excite the high frequency ultrasound transducer. Peak-to-peak voltage (*V_pp_*) and pulse duration (*t_w_*) are controlled by a function generator. Number of pulses (NP) is the total number of ultrasound pulses to target cells. The location of the transducer was controlled by a 3D translation/rotation stage. (**D**) Target cell in red is being treated by ultrasound pulse generated by high frequency ultrasonic transducer. For the intracellular delivery of macromolecules, small molecules can be presented in the same Petri dish. Yellow cone between the transducer tip and the target cell indicates ultrasound pulse (not to scale).

The 150 MHz high frequency ultrasonic transducer was developed using a conventional tran111sducer fabrication protocol [31,32]. Briefly, lithium niobate (LibNO_3_) was slapped down to 10 μm. Conductive backing layer was cast on one side of lapped LibNO_3_ before focusing. LibNO_3_ with backing layer is called acoustic stack (Figure 2A). Circular aperture was 1 mm in diameter. While heating the acoustic stack in a 90 °C oven, backing layer became soft and provided a structural integrity during the press focusing with a 2 mm diameter ball to make focal length of 1 mm (Figure 2A). The press focused acoustic stack was securely placed at the distal end of a 1.6 mm stainless-steel needle using insulation epoxy. LibNO_3_ with the thickness of 10 μm was designed to generate 150 MHz ultrasound pulse. Pulse echo was tested using a quartz target and measured center frequency was 150 MHz as shown in Figure 2B from a spectrum of echo signal. The schematic diagram depicting a system for single cell stimulation and the intracellular delivery using high frequency ultrasound is shown in Figure 2C [27]. The developed transducer (Figure 2A) was attached to a 3D translation/rotation stage to precisely locate the focus of the ultrasonic transducer for the intracellular delivery of FRET biosensors and the stimulation of target cells (Figure 2D) [27]. A function generator and a power amplifier were used to transmit an electrical pulse (EP) to trigger LibNO_3_ for ultrasound pulse generation. Peak-to-peak voltage (*V_pp_*) and pulse width (*t_w_*) were adjusted by a function generator for intracellular delivery or stimulation. Throughout the Ca^2+^ influx stimulation experiments, *V_pp_* and *t_w_* were 22 V and from 5 μs to 10 μs, respectively. 

One day before FRET live cell imaging, we delivered either FRET-GFPRed or FRET-CFPYPet using HFU. For plasmid DNA delivery using HFU, we used *V_pp_* and *t_w_* of 22 V and 20 μs, respectively [27]. FRET BS expressing cells are ready for stimulation experiments in one day.

### 2.3. Cell Culture and Reagents

HeLa cells were purchased from ATCC. Cells were cultured in Dulbecco’s Modified Eagle’s Medium supplemented with 10% FBS (Gibco, Carlsbad, CA, USA), 2 mM L-glutamine, 1 unit/mL penicillin, 100 μg/mL streptomycin, and 1 mM sodium pyruvate. Cells were cultured at 37 °C with 5% CO2. Ionomycin was purchased from Sigma. For traditional transfection, 1 μg/mL DNA plasmids were transfected into cells using Lipofectamine 3000 according to the manufacturer’s instructions (Thermo Fisher Scientific, Waltham, UK). For the intracellular delivery by HFU, 70 μg/mL DNA plasmids were used.

### 2.4. Imaging System

**Figure 3:**
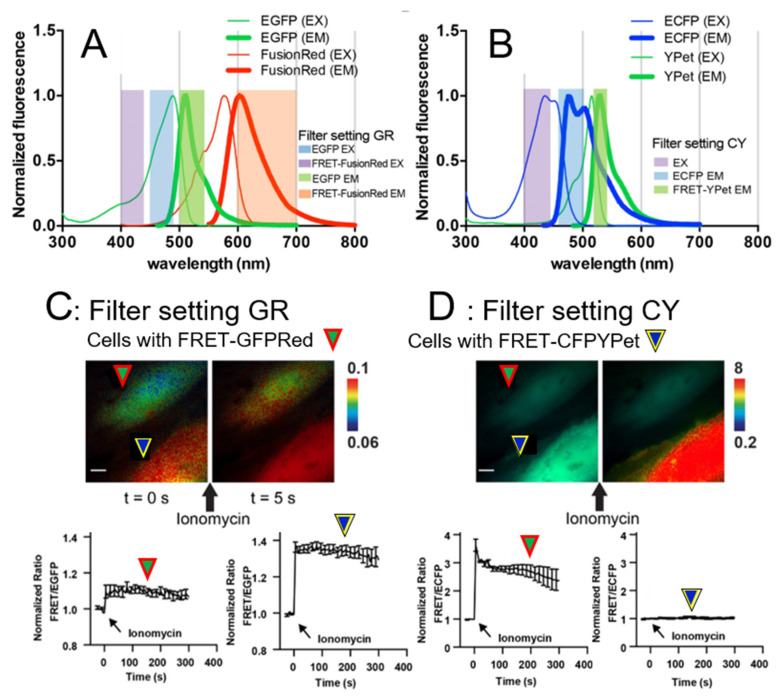
The characterization of a new FRET-based Ca^2+^ biosensor with EGFP and FusionRed fluorophores (FRET-GFPRed) by chemical stimulation. A FRET-based Ca^2+^ biosensor with ECFP and YPet (FRET-CFPYPet) was used for comparison. (**A**) Normalized fluorescence spectra showing excitation (EX, thin lines) and emission (EM, thick lines) of EGFP (green lines) and FRET-FusionRed (red lines) are presented. The filter setting GR has excitation filters for EGFP (blue region, 450–490 nm) and FRET-FusionRed (purple region, 400–440 nm) as well as emission filters for EGFP (green region, 500–550 nm) and FRET-FusionRed (red region, 600–700 nm). Time course of the typical FRET-GFPRed signal after ionomycin stimulation is presented in Figure 1B. (**B**) Normalized fluorescence spectra showing excitation (EX, thin lines) and emission (EM, thick lines) of ECFP (cyan lines) and YPet (green lines) are presented. The filter setting CY has an excitation filter (purple region, 400–450 nm) for ECFP/YPet and emission filters for ECFP (blue region, 460–500 nm) and YPet (green region, 520–550 nm). (**C**,**D**) Two Hela cells in one field of view are shown. The upper and lower cells were transfected with FRET-GFPRed and FRET-CFPYPet, respectively. The representative (**C**) FRET/EGFP and (**D**) FRET/ECFP ratio images of two cells before and after ionomycin stimulation are presented. (**C**) Time course of the FRET/EGFP signal ratio of FRET-GFPRed (left) and FRET-CFPYPet (right) indicating a spectral bleed-through of FRET-CFPYPet signal using the filter setting GR. (**D**) Time course of the FRET/ECFP signal ratio of FRET-CFPYPet (left) and FRET-GFPRed (right) indicating no spectral bleed-through of the FRET-GFPRed signal using the filter setting CY. (**C**,**D**) Normalized FRET/EGFP and FRET/ECFP signal ratios were obtained by live cell imaging using the filter settings GR and CY, respectively. Green arrowheads surrounded by red lines indicate HeLa cells transfected with FRET-GFPRed. Blue arrowheads surrounded by yellow lines represent cells transfected with FRET-CFPYPet. Black arrows represent ionomycin (chemical) stimulation. Scale bars represent 10 μm. Error bars represent +/− one SD.

The ultrasound system for the intracellular delivery and stimulation was integrated with a Nikon fluorescence microscope for FRET imaging (Figure 2C). Images were collected with a Nikon microscope and a cooled charge-coupled device (CCD) camera using the MetaFluor 6.2 and MetaMorph software packages (Universal Imaging, Downing town, PA, USA). The filter setting GR in Figure 3A for EGFP/FusionRed pair consisted of EGFP EX 470/40 and EGFP EM 525/50 combined with EGFP dichroic 495 and FRET-FusionRed EX 420/40 and FRET-FusionRed EM 650/100 combined with FRET-FusionRed dichroic 595. The exposure time of FRET-FusionRed was five times longer than that of EGFP. The filter setting CY for ECFP/YPet pair in Figure 3B contained EX 420/40, ECFP EM 480/40, and YPet EM 535/30 combined with dichroic 455. The RFP filter set consisted of EX 560/40 and EM 630/75 combined with dichroic 585 to distinguish cells transfected with FRET-GFPRed or FRET-based Ca^2+^ BS with ECFP and YPet (FRET-CFPYPet). We collected five channels sequentially using filter setting CY and GV. As shown in Figure 3A,B, acquired channel data are EX-ECFP EM of filter setting CY for channel 1, EX-(FRET-YPet EM) of filter setting CY for channel 2, EGFP EX-EGFP EM of filter setting GR for channel 3, EGFP EX-(FRET-FusionRed EM) of filter setting GR for channel 4, and differential interference contrast (DIC) for channel 5. A neutral density filter was used to control excitation light intensity. The fluorescence intensities of non-transfected cells were quantified as background signals and were subtracted from the donor and acceptor signals detected from the transfected cells. MetaFluor was used to calculate the pixel-by-pixel ratio images of FRET/ECFP and FRET/EGFP based on the background-subtracted fluorescence intensity images and calculate standard deviation (SD) and standard error of the mean (SEM) of FRET responses. The emission ratio images were shown in the intensity modified display (IMD) mode [19].

## 3. Results

### 3.1. Characterization of a New FRET Biosensor with EGFP and FusionRed Expressed in Mammalian Cells

We have developed a new FRET-based Ca^2+^ biosensor (BS) using a new fluorophore pair by combining EGFP and FusionRed (FRET-GFPRed). FRET-GFPRed requires filter settings different from those of the typical FRET-based Ca^2+^ BS with ECFP and YPet (FRET-CFPYPet) [20,22]. FRET-GFPRed has a central biosensor piece containing Calmodulin (CaM), a flexible linker peptide, and M13, which are concatenated between EGFP and FusionRed (Figure 1A). Figure 1B indicates ionomycin stimulation using FRET-GFPRed and we used filter setting GR in Figure 3A. The spectral overlap between EGFP and FusionRed is shown in Figure 3A. The FRET-CFPYPet spectra between ECFP and YPet and the microscope filter setting CY are shown in Figure 3B. The quantum yields of EGFP and FusionRed are 0.60 and 0.19, respectively, and the extinction coefficients of EGFP and FusionRed are 56 mM^−1^cm^−1^ and 94.5 mM^−1^cm^−1^, respectively [23,33]. Relative brightness of FusionRed to EGFP is 53% (quantum yield multiplied by extinction coefficient). We speculated that the exposure time of FusionRed should be longer than that of EGFP for proper FRET-FusionRed/EGFP ratio imaging. We tested this hypothesis to optimize the filter setting GR for FRET-GFPRed by acquiring FRET-FusionRed/EGFP ratio at three filter settings (Supplementary Figure S1). The optimized filter setting GR was found to be EGFP EX 470/40, EGFP EM 525/50 combined with dichroic 495 and FRET-FusionRed EX 470/40, FRET-FusionRed EM 650/100 combined with dichroic 595 (Figure 3A). The exposure time ratio of the FRET-FusionRed channel to the EGFP channel using the filter setting GR was five. FRET-GFPRed was transfected into HeLa cells and stimulated with ionomycin to induce a FRET response due to increased levels of intracellular calcium [34]. FRET-GFPRed consistently exhibited a 10–15% increase in FRET-FusionRed/EGFP ratio using the filter setting GR, as shown in Figure 1B.

### 3.2. Spectral Compatibility of FRET-GFPRed and FRET-CFPYPet for Dual-FRET Imaging of Intercellular Cell Signaling

We have investigated the spectral compatibility of FRET-GFPRed and FRET-CFPYPet using the filter settings GR and CY (Figure 3A,B). FRET-GFPRed and FRET-CFPYPet were individually transfected into HeLa cells, and then seeded together for live cell imaging. We found two adjacent cells, which expressed FRET-GFPRed (green arrowhead surrounded by red lines in Figure 3C,D) and FRET-CFPYPet (blue arrowhead surrounded by yellow lines in Figure 3C,D). The two cells transfected with FRET-GFPRed and FRE-CFPYPet were easily distinguished using the RFP channel, as shown in Supplementary Figure S2. In one window, we could simultaneously monitor distinct FRET signals from two neighboring cells with high accuracy and specificity (Figure 3C,D). The filter settings GR and CY were tested by simultaneously stimulating two cells with ionomycin. The cell indicated by green arrowhead surrounded by red lines in Figure 3C presents significant FRET changes from FRET-GFPRed biosensor. These two images were generated with the emission ratio of 0.06–0.1. The FRET-CFPYPet signal changed noticeably when the filter setting CY and the emission ratio of 0.2–8 were used as indicated by blue arrowhead surrounded by yellow lines in Figure 3D.

### 3.3. Unique Dual FRET Imaging with Single Cell Stimulation Using High Frequency Ultrasound

**Figure 4:**
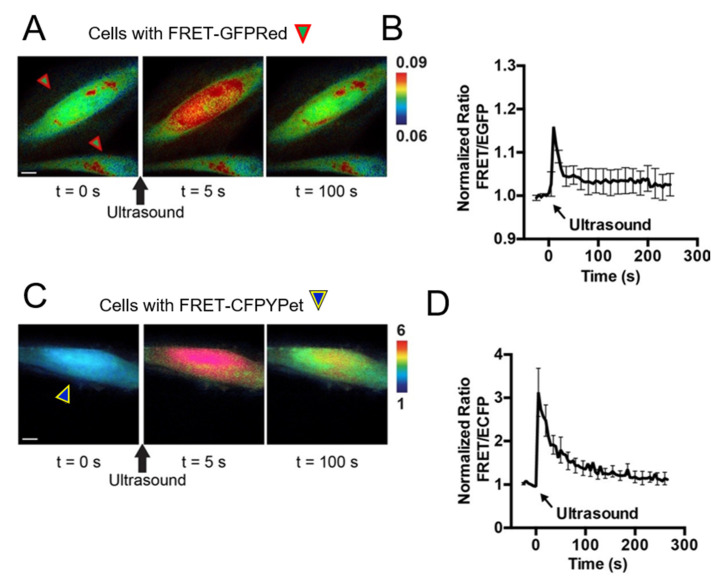
A new FRET-based Ca^2+^ biosensor with EGFP and FusionRed fluorophores (FRET-GFPRed) stimulated with high frequency ultrasound. A FRET-based Ca^2+^ biosensor with ECFP and YPet (FRET-CFPYPet) was used for comparison. FRET-GFPRed and FRET-CFPYPet were transfected by Llipofectamine 3000. (**A**) Representative FRET/EGFP ratio images of FRET-GFPRed expressed HeLa cells before (t = 0 s), 5 s, and 100 s after ultrasound stimulation. FRET images were taken using the filter setting GR as shown in Figure 3A. A high frequency ultrasound was used to target only the upper cell for ultrasound stimulation. The lower cell was not stimulated. (**B**) Time course of the FRET/EGFP signal ratio of FRET-GFPRed after ultrasound stimulation is presented. (**C**) Representative FRET/ECFP signal ratio images of FRET-CFPYPet before (t = 0 s), 5 s, and 100 s after ultrasound stimulation of HeLa cells are presented. FRET images were taken using the filter setting CY as shown in Figure 3B. (**D**) Time course of the FRET/ECFP signal ratio of FRET-CFPYPet after ultrasound stimulation is presented. Green arrowheads surrounded by red lines indicate HeLa cells transfected with FRET-GFPRed. Blue arrowheads surrounded by yellow lines indicate cells transfected with FRET-CFPYPet. Black arrows indicate ultrasound stimulation. The duration of the ultrasound stimulation was from 5 to 10 μs. Scale bars represent 10 μm. Error bars represent +/− one SEM.

**Figure 5:**
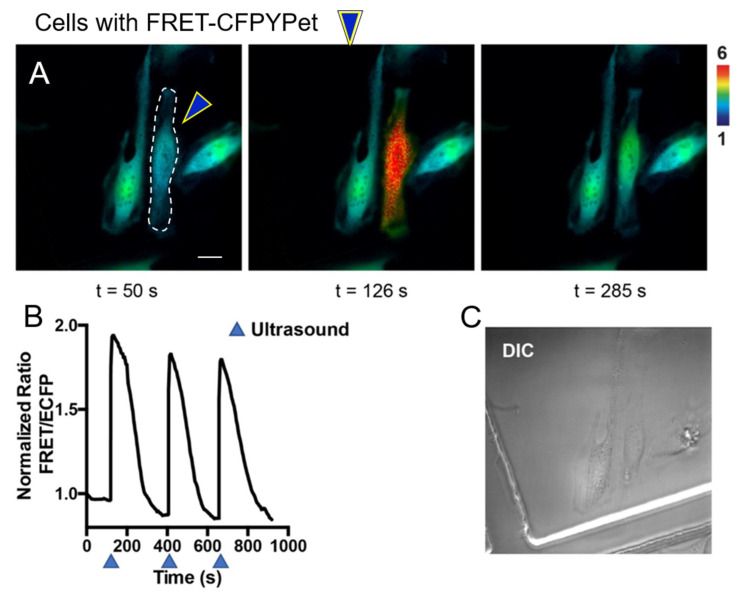
Repeated and transient HFU stimulation. (**A**) Images and (**B**) time course of FRET/ECFP signal ratio of Ca^2+^ biosensor show transient and repeated stimulations by high-frequency ultrasound at the target single cell (dashed line in **A**). (**B**) High-frequency ultrasound (arrowheads) was applied three times for 5 μs. (**C**) A DIC image of cells in (**A**).

Chemical stimulations using pervanadate, ionomycin, and epidermal growth factor are considered the gold standard compounds to study intra- and intercellular molecular events using FRET-based biosensors by live cell imaging. We introduce a new stimulation strategy by using HFU. Unlike chemical stimulation, HFU provides single cell level stimulation because of HFU’s focusing capability. HeLa cells were transfected with FRET-GFPRed (Figure 4A) and FRET-CFPYPet (Figure 4C) with Lipofectamine 3000. The transfection efficiency of Lipofectamine 3000 was approximately 60–80%. HFU was applied to a target cell and FRET/EGFP signal ratio changes were observed only in the targeted cells (Figure 4A,B). Figure 4C,D present FRET changes of FRET-CFPYPet biosensor under HFU stimulation. Transient and repeated stimulations can also be achieved for longitudinal monitoring of cell responses using HFU stimulation (Figure 5). When HFU introduces Ca^2+^ into the cytoplasm of cells, FRET BS immediately detects the increase of intracellular Ca^2+^ concentration, which activates the plasma membrane Ca^2+^ ATPase (PMCA). PMCA starts pumping out Ca^2+^ to maintain cell’s metabolism [35]. Calcium ion channels may contribute for FRET increase upon HFU stimulation; however, because applied level of *V_pp_* and *t_w_* induces cell membrane disruption, the influx of Ca^2+^ through calcium ion channel may not be significant. Additionally, use of HFU for stimulating cells eliminates the needs for washing out chemicals during live cell imaging.

### 3.4. Making Cell Pairs for Unique Dual FRET Imaging Using High Frequency Ultrasound

**Figure 6:**
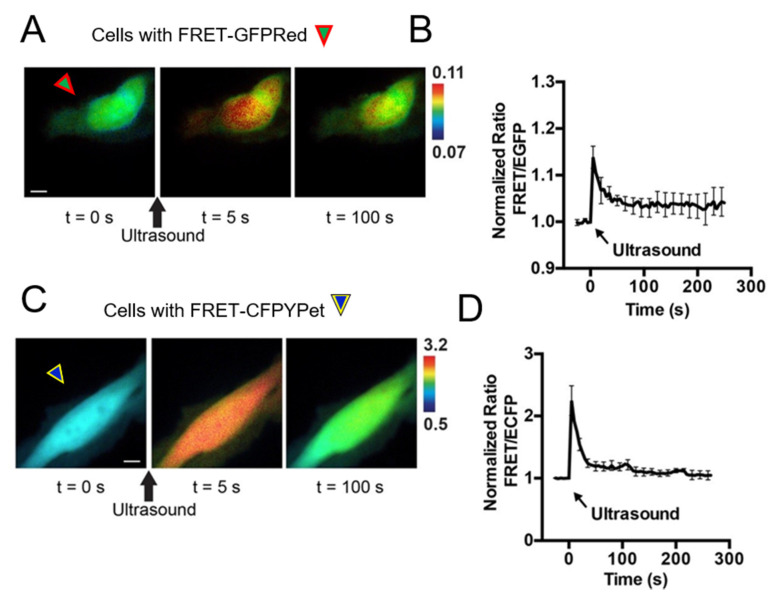
A new FRET-based Ca^2+^ biosensor with EGFP and FusionRed fluorophores (FRET-GFPRed) stimulated with high frequency ultrasound. A FRET-based Ca^2+^ biosensor with ECFP and YPet (FRET-CFPYPet) was used for comparison. FRET-GFPRed and FRET-CFPYPet were transfected by high frequency ultrasound, which can specifically transfect single cells with the desired FRET biosensor. (**A**) Representative FRET/EGFP signal ratio images of FRET-GFPRed in a HeLa cell before (t = 0 s), 5 s, and 100 s after ultrasound stimulation are presented. FRET images were taken using the filter setting GR as shown in Figure 3A. (**B**) Time course of the FRET/EGFP signal ratio of FRET-GFPRed after ultrasound stimulation is presented. (**C**) Representative FRET/ECFP signal ratio images of FRET-CFPYPet before (t = 0 s), 5 s, and 100 s after ultrasound stimulation is shown. FRET images were taken using the filter setting CY as shown in Figure 3B. (**D**) Time course of the FRET/ECFP ratio of the FRET-CFPYPet after ultrasound stimulation (t = 0) is presented. Green arrowheads surrounded by red lines indicate HeLa cells transfected with FRET-GFPRed. Blue arrowheads surrounded by yellow lines represent cells transfected with FRET-CFPYPet. Black arrows indicate ultrasound stimulation. The duration of the stimulation was from 5 to 10 μs. Scale bars represent 10 μm. Error bars represent +/− one SEM.

In addition to the stimulation of single cells using HFU, we also used HFU to deliver FRET biosensors into individual cells [26,27]. In the current study, FRET-GFPRed and FRET-CFPYPet were transfected into target cells individually by HFU and HFU stimulates one single cell at a time. The intracellular delivery efficiency by HFU was maintain to at least 85% [27]. The advantage of transfection by HFU is that we can deliver different FRET-based BS into desired cells with high spatial resolution and can precisely define cell pairs to study cell–cell interactions. As an example, FRET-GFPRed and FRET-CFPYPet were transfected into two different cells using HFU. In Supplementary Figure S2, only the cell at the lower left corner could be observed using the RFP channel due to fluorescence of FusionRed from FRET-GFPRed. FRET images of FRET-GFPRed (Figure 6A) and FRET-CFPYPet (Figure 6C) of HeLa cells demonstrate that both the intracellular delivery and stimulation using HFU were successfully performed. The changes in FRET signal shown in Figure 6B,D indicate a transient and immediate increase in signal right after HFU stimulation. Similar to stimulation with ionomycin, spectral bleed-through of FRET-CFPYPet using the filter setting GR was observed with HFU stimulation (Supplementary Figures S3 and S4).

## 4. Discussion

We have developed a FRET-based Ca^2+^ biosensor with EGFP and FusionRed fluorophore pair (FRET-GFPRed) for dual FRET live cell imaging. We chose FusionRed as the acceptor of the new FRET pair because it is a monomeric and less toxic red fluorescent protein [23]. The new FRET-GFPRed has fast response kinetics upon the introduction of Ca^2+^.

FRET-GFPRed shows 10–15% FRET changes and FRET-CFPYPet changed 200–300% upon impulse-like HFU, as CFP-YPet pair has been the most efficient FRET pair. The innovation here is the spectrally distinguishable FRET pairs under HFU for multimolecular live cell imaging.

A spectral bleed-through of FRET-GFPRed and FRET-CFPYPet biosensors existed at some degree when FRET changes were imaged using filter setting CY and GR (Figure 3A,B and Supplementary Figures S3 and S4). Filter setting GR and CY are optimized filter settings for FRET-GFPRed and FRET-CFPYPet biosensors, respectively (Figure 3). Physically, we optimized filter settings to acquire appropriate fluorescent signals from donors (EGFP for FRET-GFPRed and ECFP for FRET-CFPYPet) and acceptors (FusionRed for FRET-GFPRed and YPet for FRET-CFPYPet). Because there are some spectral overlaps between two biosensors, which are the main causes of spectral bleed-through, we have found a way to utilize the donor/FRET ratio value for distinguishing different FRET biosensors. The ratio of signal intensities between FusionRed and EGFP represents FRET signal of FRET-GFPRed. For FRET-CFPYPet biosensor, FRET signal is the ratio between YPet and ECFP. As mentioned earlier, because the relative brightness of EGFP is twice as bright as that of FusionRed, the initial FRET signal of FRET-GFPRed is insignificant compared to unity. However, the FRET signal of FRET-CFPYPet is close to unity because the relative brightness of ECFP and YPet are comparable. Then, FRET images of FRET-GFPRed and FRET-CFPYPet can be differentiated using different scales of emission ratio as shown in Figure 3C,D, Figure 4 and Figure 6. To support this argument, when the emission ratio is between 0.06 and 0.1, FRET-GFPRed clearly shows FRET changes of the cell indicated by green arrowhead with red lines in Figure 3C. Images with the emission ratio between 0.2 and 8 clearly show FRET increase only from the cell with FRET-CFPYPet, which is indicated by blue arrowhead with yellow lines in Figure 1F. Before live cell, imaging is started, cells with two different FRET biosensors can also be differentiated by using RFP channel (Supplementary Figure S2). Therefore, the developed FRET-GFPRed can visualize intracellular molecular signals with unique fluorescent spectra different from the commonly used FRET biosensors with ECFP and YPet fluorophore.

Specific applications of dual FRET-based live cell imaging with high frequency ultrasound (HFU) as a delivery and stimulation tool may be the visualization of two molecular events during stem cell reprogramming and the investigation of mechanotransduction ion channel in the future. We have defined HFU as a new technique to mechanically stimulate single cells to append precise spatial resolution and repeated simulation for a longitudinal monitoring of single cells to FRET-based live cell imaging. Pairs of cells for cell–cell interaction studies can be precisely established when different FRET-based biosensors are delivered into the desired cells using HFU. HFU stimulates a single cell without affecting the surrounding cells, triggering multiple signaling responses on the targeted cells while simultaneously monitors the neighboring cells. Thus, HFU may be used for cell–cell interaction studies at single cell level. HFU’s single cell targeting capability may be also used to investigate intercellular calcium wave propagation [36,37]. A fast response, significant membrane poration, and slow stretch-activated ion channels were identified from ultrasound-induced microbubble oscillation [38]. In this paper, we investigated FRET response at single cell level using HFU with the center frequency of 150 MHz. After the technique improves to develop ultrahigh-frequency transducers with the center frequency of over 500 MHz, we envision that we can manipulate and stimulate organelles in a cell. We can directly manipulate endoplasmic recticulum or mechanotransduction ion channels and visualize the Ca^2+^ transport using FRET biosensor, which will answer important physiological and pathological problems. A feedback control mechanism to locate the focus of these types of ultra-high frequency transducer will be increasingly appreciated. The new FRET-GFPRed has limitations as well. First, FRET efficiency needs to be improved, which may be achieved by changing the order and the distance of the fluorophores. Second, we need to clone this fluorophore pair to other FRET BS such as Src and Fyn for multiplexed FRET live cell imaging.

A new FRET-based Ca^2+^ biosensor with an EGFP and FusionRed fluorophore pair was developed to distinguish FRET signals from typical ECFP/YPet based FRET biosensors. High-frequency ultrasound was introduced for the intracellular delivery of FRET biosensors and stimulation of single cells with improved spatiotemporal resolution. Unlike chemical stimulation, high-frequency ultrasound stimulation does not require washout steps between stimulations. Herein, we demonstrated that different types of FRET biosensors were delivered into targeted single cells by high frequency ultrasound and the developed FRET Ca^2+^ biosensor exhibited a transient and significant Ca^2+^ signal response under high frequency ultrasound stimulation. Therefore, this FRET biosensor combined with high frequency ultrasound-based intracellular delivery and cell stimulation can be useful for establishing cell pairs in cell–cell interaction studies. We further expect that specific targeting of FRET biosensors and high frequency ultrasound stimulation of subcellular structures and organelles will stimulate new avenues of research in cell biology.

We will further describe the proposed technique as a future endeavor. Specific applications of dual FRET-based live cell imaging with HFU as a delivery and stimulation tool may be the visualization of two molecular events during stem cell reprogramming and the investigation of mechanotransduction ion channel.

## Supplementary Materials

The following are available online at https://www.mdpi.com/1424-8220/20/17/4998/s1, Figure S1: The optimization of filter setting GR (Figure 3A) for FRET-GFPRed. EGFP EX 470/40 and EGFP EM 525/50 combined with dichroic 495 were used for all case. Three FRETFusionRed filter sets were tested. Time courses of the FRET-FusionRed/EGFP signal ratio of FRET-GFPRed after ionomycin stimulation (t = 0 s) were imaged with (A) FRET-FusionRed EX 470/30 and FRET-FusionRed EM 650/100 combined with same exposure time for EGFP and FRET-FusionRed, (B) FRET-FusionRed EX 470/30 and FRET-FusionRed EM 650/100 combined with five times longer FRET-FusionRed exposure time than EGFP exposure time, and (C) FRETFusionRed EX 420/40 and FRET-FusionRed EM 650/100 combined with five times longer FRETFusionRed exposure time than EGFP exposure time. The optimized filter set for FRETFusionRed/EGFP was found to be the third filter set as shown in (C). This filter set was named as the filter setting GR (Figure 3A) and was used to image FRET-GFPRed biosensors, Figure S2: Left panel shows a HeLa cell expressing FRET-GFPRed (green arrowhead surrounded by red lines) and right panel shows a HeLa cell transfected with FRETCFPYPet (blue arrowhead surrounded by yellow lines). Two cells were sequentially transfected with FRET-GFPRed and FRET-CFPYPet by intracellular delivery using high frequency ultrasound. These two cells demonstrate that high frequency ultrasound can make a pair of cells with desired FRET-based biosensors. FusionRed in FRET-GFPRed are distinctively imaged by RFP channel. A scale bar indicates 20 μm, Figure S3: (A) A bleed-through in the filter setting GR shows the changes in time course plot of FRET/EGFP signal ratio of FRET-CFPYPet transfected cells. (B) No bleed-through is observed in the FRET/ECFP signal ratio of FRET-GFPRed transfected cells under filter setting CY. FRET biosensors were transfected by Lipofectamine 3000. Ultrasound was applied at t = 0 for 5–10 μs. Error bars indicate ± one SEM, Figure S4: (A) A bleed-through in the filter setting GR shows the changes in time course plot of FRET/EGFP signal ratio of FRET-CFPYPet transfected cells. (B) No bleed-through is observed in the FRET/ECFP signal ratio of FRET-GFPRed transfected cells under the filter setting CY. FRET biosensors were transfected by high frequency ultrasound. Ultrasound was applied at t = 0 for 5–10 μs, Supplementary Video 01: Video recording of Figure 4A. Two cells were transfected with FRETGFPRed. The cell on top was stimulated by high frequency ultrasound. Intensity of the video were not modified, Supplementary Video 02: Video recording of Figure 5. Three cells were transfected with FRETCFPYPet. The cell in the middle was stimulated by high frequency ultrasound three times. Intensity of the video were not modified.
